# Characterization of *Candida* species isolated from vulvovaginal candidiasis by MALDI-TOF with *in vitro* antifungal susceptibility profiles

**DOI:** 10.18502/cmm.7.4.8405

**Published:** 2021-12

**Authors:** Narges Aslani, Roya Kokabi, Fatemeh Moradi, Kiana Abbasi, Narges Vaseghi, Mohammad Hosein Afsarian

**Affiliations:** 1 Infectious and Tropical Diseases Research Centre, Tabriz University of Medical Sciences, Tabriz, Iran; 2 Department of Obstetrics and Gynecology, School of Medicine, Fasa University of Medical Sciences, Fasa, Iran; 3 Department of Medical Mycology and Parasitology, School of Medicine, Fasa University of Medical Sciences, Fasa, Iran; 4 Department of Microbiology, Zanjan Branch, Islamic Azad University, Zanjan, Iran; 5 Department of Pathobiology, Science and Research Branch, Islamic Azad University, Tehran, Iran; 6 HIV/AIDS Research Center, Fasa University of Medical Sciences, Fasa, Iran

**Keywords:** Antifungal susceptibility, *Candida* species, MALDI-TOF, Vulvovaginal candidiasis

## Abstract

**Background and Purpose:**

Vulvovaginal candidiasis (VVC) is an opportunistic infection due to *Candida* species, one of the most common genital tract diseases among reproductive-age women. The present study aimed to investigate the prevalence of VVC among non-pregnant women and identify the epidemiology of the involved *Candida* species with the evaluation of antifungal susceptibilities.

**Materials and Methods:**

Matrix-assisted laser desorption/ionization time-of-flight mass spectrometry (MALDI-TOF MS) was performed to identify *Candida* species isolated from the genital tract of 350 non-pregnant women. Moreover, antifungal susceptibility testing was performed according to the Clinical and Laboratory Standards Institute broth microdilution method guidelines (M27-A3 and M27-S4).

**Results:**

Vaginal swab cultures of 119 (34%) women yielded *Candida* species. *Candida albicans* was the most frequently isolated species (68%), followed by *Candida glabrata* (19.2%). Voriconazole was the most active drug against all tested isolates showing an MIC50/MIC90 corresponding to 0.016/0.25 µg/mL, followed by posaconazole (0.031/1 µg/mL). Overall, resistance rates to fluconazole, itraconazole, and voriconazole were 2.4%, 4.8% and, 0.8% respectively. However, posaconazole showed potent *in vitro* activity against all tested isolates.

**Conclusion:**

Results of the current study showed that for the effectual therapeutic outcome of candidiasis, accurate identification of species, appropriate source control, suitable antifungal regimens, and improved antifungal stewardship are highly recommended for the management and treatment of infection with *Candida*, like VVC.

## Introduction

Abnormal growth of different *Candida* species in the genital tract of the female leads to an infection called vulvovaginal candidiasis (VVC). It is estimated that VVC, as an inflammatory disease of the vulva and vagina, is the second most frequent vaginal infection after bacterial vaginosis [ 1 - 3 ]. The signs and symptoms of VVC include vulvar pruritus, vaginal itching, abnormal curd-like vaginal discharge, irritation, burning sensation, pain during intercourse, and vaginal erythema [ 4 ].

Common predisposing risk factors of VVC comprise of socio-demographic characteristics, pregnancy, uncontrolled diabetes mellitus, oral contraceptives, sexual activity, extensive use of broad-spectrum antibiotics, poor personal hygiene, and specific immunological defect. Overall, the reasons for the global importance of the VVC are the high frequency of occurrence, sexually transmitted infections, ascending genital tract infections, and direct and indirect economic costs [ 5 , 6 ].

Several studies reported that *Candida albicans* is predominantly involved in VVC, followed by *Candida glabrata*, *Candida tropicalis*, *Candida parapsilosis*, and *Pichia kudriavzevii* (*Candida krusei*) [ 4 , 6 , 7 ]. Nevertheless, the increasing prevalence of non-*albicans Candida* species with a reduced susceptibility acquired resistance or intrinsic resistance to the antifungal drugs currently administered has become the most important issue of treatment failure, over the past decade [ 4 , 8 ]. Therefore, appropriate and precise identification of *Candida* to the species level and determination of their drug susceptibility patterns to azole compounds as the most commonly used class of drug agents can be useful for the provision of effective treatment of *Candida* infections. 

With this background in mind, the purpose of the current study was to identify *Candida* species responsible for VVC among non-pregnant women using Matrix-assisted laser desorption/ionization time-of-flight mass spectrometry (MALDI-TOF MS) as a reliable and rapid technique for the identification of cryptic species. Additionally, the isolates were subjected to *in vitro* antifungal susceptibility profiles via microdilution broth.

## Materials and Methods

During 2 years, 350 samples among immunocompetent non-pregnant women have been selected to examine for vaginal secretion, considering the presence of vulvovaginitis symptoms suggestive of vaginitis, including vulvar burning, pruritus vulvae, dyspareunia, vaginal soreness and irritation, pain or discomfort during urination, and abnormal vaginal discharge referred to the department of obstructive and gynecology at the Fasa Valiasr Hospital, Fasa, Iran.

All samples were obtained from the posterior fornix of the vagina with sterilized vaginal swabs and initially examined by gram stain, followed by inoculation on malt extract agar (Difco) supplemented with chloramphenicol (50 mg/ml), CHROMagar *Candida* medium (CHROMagar Company, Paris, France) to ensure purity, and incubated at 35-37 °C for 48 h [ 7 ]. Approval of the research was acquired from the Research Ethics Committee of the Fasa University of Medical Sciences (IR.FUMS.REC.1395.85), and written consent was obtained from all patients involved.

The MALDI-TOF MS-based identification of all isolates to the species level was performed according to Bruker Daltonics (Biotyper RTC software, version 3.0 (Bruker Daltonics, Bremen, Germany)) using the ethanol (EtOH)/ formic acid (FA) extraction protocol [ 9 , 10 ]. In addition, discrimination of the *C. albicans* species complex, i.e., *C. albicans*, *C. dubliniensis*, *C. africana*, and *C. stellatoidea* was performed by the amplification of the hyphal wall protein 1 (HWP1) gene as previously described [ 11 , 12 ].

*In vitro* antifungal susceptibility was performed for identified isolates according to the recommendations in the Clinical and Laboratory Standards Institute broth microdilution method (CLSI) reference guidelines M27-A3 and M27-S4 [ 13 , 14 ]. Antifungal drugs tested were fluconazole, itraconazole, voriconazole, and posaconazole (All, Sigma-Aldrich, Germany). *P. kudriavzevii* ATCC 6258 and *C. parapsilosis* ATCC 22019 were utilized as quality control for all antifungal susceptibility tests.

## Results

In total, 125 (35.7%) strains of *Candida* were isolated from vaginal secretion samples collected from 350 non-pregnant women with signs or/and symptoms of vaginal infection. The mean age of the patients with a positive culture for *Candida* species was 35.8 years, ranging from 20 to 52 years (n=2 <20 and n=5 >52). Vulvovaginal itching (71%), vaginal discharge (49%), vulvovaginal burning sensation (34%), and pain (17%) were among the most common described signs and symptoms. However, recurrent vulvovaginal candidiasis was not obtained among women in the present study. 

The results of identification based on the conventional method (CHROMagar) were confirmed by the MALDI-TOF assessment. Conventional method and MALDI-TOF assessment identified 125 isolates as *C. albicans* (n=86), *C. glabrata* (n=24), *P. kudriavzevii* (n= 8), *Cyberlindnera fabianii* (previously *Hansenula fabianii*, *Pichia fabianii*, and *Lindnera fabianii*, n=4), *Kluyveromyces marxianus* (*Candida kefyr*, n=2), and *C. parapsilosis* (n=1). 

**Figure 1:**
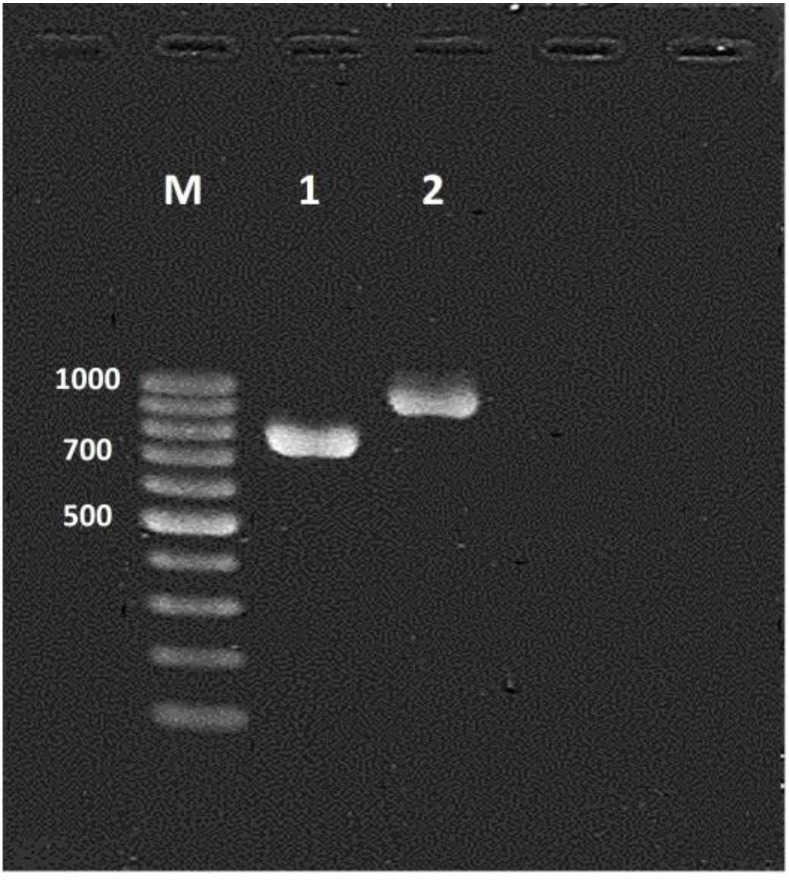
Species specific amplification of the *hwp1* gene; Lane 1 for *C. Africana* (~ 740 bp); Lane 2 for *C. albicans* (~ 941 bp); Lane M, a molecular size marker

However, PCR amplification of the hwp1 gene was performed for 86 of *C. albicans* which has been identified by MALDI-TOF, and only one species was recognized as *Candida africana* (n=1). This was confirmed by DNA sequencing assessment (Figure 1. Supplementary). It is noteworthy all four strains of *C. fabianii* strains were reconfirmed using a dual-function PCR as well [ 15 ]. It is noteworthy that MALDI-TOF is unable to robustly distinguish species belonging to *C. albicans* complexes, such as *C. dubliniensis*, *C. africana*, and *C. stellatoidea*. 

Table 1 summarizes the MIC50, MIC90 and geometric mean MIC (GM) for each species. In total, 125 isolates of *Candida* species were analyzed for their susceptibility to fluconazole, itraconazole, voriconazole, and posaconazole. The uniform patterns of low MIC ranges in all clinical strains were voriconazole (0.016-1 µg/mL), posaconazole (0.016-2 µg/mL), itraconazole (0.016-8 µg/mL), and fluconazole (0.125-64 µg/mL) in increasing order. In terms of MIC50/MIC90, voriconazole was the most active drugs against all isolates (0.016/0.25 µg/mL) followed by posaconazole (0.031/1 µg/mL), itraconazole (0.063/2 µg /mL), and fluconazole (0.5/16 µg /mL).

**Table 1:** *In vitro* susceptibility testing of 125 VVC clinical isolates of *Candida* species to four triazole antifungal agents (MIC: minimum inhibitory concentration range, geometric (G) mean, MIC50, and MIC90 values are expressed in µg/ml)

Strains (no.) and Antifungal drugs	S	SDD	R	Range MICs (μg/ml)	MIC50 / MIC90 (μg/ml)	Mode	G mean
*Candida albicans* (n=85)							
*FLC*	80	2	3	0.125-32	0.25/0.5	0.25	0.36
*ITC*	77	4	4	0.016-8	0.063/0.125	0.063	0.05
*VRC*	81	3	1	0.016-1	0.016/0.016	0.016	0.01
*POS*	80	1	4	0.016-1	0.016/0.125	0.016	0.03
*Candida glabrata* (n=24)							
*FLC*	-	24	-	2-32	4/16	4	5.82
*ITC*	4	14	6	0.25-4	1/4	1	1.09
*VRC*	24	-	-	0.031-0.5	0.063/0.25	0.063	0.09
*POS*	18	4	2	0.25-4	0.5/2	0.5	0.53
*Pichia kudriavzevii* (n=8)							
*FLC*	-	-	8	16-64	-	-	-
*ITC*	3	2	3	0.25-1	-	-	-
*VRC*	8	-	-	0.125-0.25	-	-	-
*POS*	7	1	-	0.125-1	-	-	-
*Cyberlindnera fabianii* (n=4)							
*FLC*	2	1	1	0.25-16	-	-	-
*ITC*	1	-	3	0.016-2	-	-	-
*VRC*	4	-	-	0.016-0.125	-	-	-
*POS*	1	2	1	0.031-1	-	-	-
*Kluyveromyces marxianus* (n=2)							
*FLC*	1	1	-	0.5-4	-	-	-
*ITC*	1	-	1	0.25-2	-	-	-
*VRC*	2	-	-	0.016-0.063	-	-	-
*POS*	2	-	-	0.125-0.5	-	-	-
*Candida africana* (n=1)							
*FLC*	-	1	-	4	-	-	-
*ITC*	-	-	1	2	-	-	-
*VRC*	1	-	-	0.125	-	-	-
*POS*	-	-	1	1	-	-	-
*Candida parapsilosis* (n=1)							
*FLC*	-	-	1	8	-	-	-
*ITC*	-	-	1	1	-	-	-
*VRC*	-	1	-	0.5	-	-	-
*POS*	-	-	1	1	-	-	-

VRC: Voriconazole; FLC: Fluconazole; ITC: Itraconazole; POS: Posaconazole

The results showed the widest range and highest MICs of *Candida* species were observed for fluconazole (0.125-64 µg/ml). In total, 80 *C. albicans* (94.2%) were sensitive, 2 (2.3%) were susceptible-dose-dependent (SDD), and 3 (3.5%) were resistant to fluconazole. However, all of the *C. glabrata*, the second common isolates, were SDD to ﬂuconazole. Notably, a single clinical isolate of *C. parapsilosis* was resistant to ﬂuconazole and two isolates of *C. fabianii*, a rare and uncommon *Candida* species, showed high fluconazole MIC (4 and 16 μg/ml). 

All clinical isolates of *P. kudriavzevii* were intrinsically considered resistant to fluconazole; however, their breakpoint was not provided by CLSI guidelines. Nevertheless, all of *P. kudriavzevii* isolates were susceptible to voriconazole and posaconazole. Notably, three (37.5%) of the *P. kudriavzevii* were resistant to itraconazole. Moreover, resistance to voriconazole was observed among *C. albicans* isolates 1 (1.2%), while 81 (95.3%) of the *C. albicans* and 8 (100%) of the *P. kudriavzevii* isolates were susceptible. Remarkably, three (3.5%) of the *C. albicans* and single clinical isolate of the *C. parapsilosis* isolates were SDD to voriconazole. Susceptibility of *C. albicans* with respect to itraconazole was analyzed as follows: itraconazole sensitivity rate was in 77 (90.6 %), SDD in 4 (4.7%), and resistance in 4 (4.7%) of isolates. 

*Candida albicans* complex isolate (*C. africana*) showed susceptiblity to voriconazole; however, it was SDD to fluconazole and resistant to itraconazole and posaconazole. Overall, in terms of geometric means (GM) MICs, voriconazole was the most active agent against all isolates (n=125), followed by posaconazole in comparison with itraconazole and fluconazole. Moreover, GM elevated of *C. glabrata* was observed for fluconazole (5.82 μg/ml) in comparison to voriconazole (0.01 μg/ml) in *C. albicans*. Both *K. marxianus* isolates were found to have low MICs to voriconazole, while one isolate was resistant to itraconazole and SDD to fluconazole and posaconazole. However, another isolate was shown to be SDD to only itraconazole.

## Discussion

The correct and precise identification of *Candida* species and evaluation of their drug susceptibility profiles provide helpful information for the effectual therapeutic outcome and cause a remarkable economic impact on the public health system. We investigated the distribution and susceptibility profiles of 125 *Candida* species obtained from patients with VVC in Fasa, Iran. The prevalence rates of VVC in the present study (41.6%) among women within the age rang e of 30-39 years old had differences, compared to a recent study that reported the highest VVC rates in the age range of 18-29 years old in Greek women [ 6 ]. The data are shown in our study consistent with a previously published report from Iran epidemiological survey comprising 559 patients [ 16 ].

The findings of the current study revealed that the prevalence rate of *Candida* vaginitis among non-pregnant women was 34% (119/350). However, in some studies, such as the one performed by Mushi et al. in Tanzania, 65.6% of the pregnant women were affected with *Candida* vaginitis (197/300) [ 3 ]. The frequency of VVC varied from 5.4% to 60% and 8.2%-75% in reports from different regions of the world as well as Iran, respectively [ 17 - 27 ]. Overall, this different frequency of VVC around the world may be related to social and cultural factors, hygiene customs, locations, population analyzed, and diagnostic techniques [ 28 ] in such studies.

Among the seven species identified by MALDI-TOF MS in the current study, *C. albicans* was the predominant one accounting for 68% (85/125) isolates obtained from vaginal secretions. This finding was in concordance with the studies of Ghajari *et al*. (67.7%) and Bonyadpour et al. (66.6%) [ 16 , 29 ]. The highest recovery rates of *C. albicans* in patients with VVC have been reported in Iran (88.2%) [ 25 ].

In the current study, non-*albicans Candida* species were isolated from 32% (40/125) of the suffering women, which was in agreement with earlier studies [ 29 , 30 ]. Nevertheless, several recent studies reported comparatively higher recovery rates of VVC caused by non-*albicans Candida*, which are less susceptible or resistant to currently administered antifungals than common Candida species [ 4 , 18 , 31 - 34 ].

In the present study, *C. glabrata* was the most common among non-*albicans Candida* species followed by *P. kudriavzevii*. Moreover, *C. glabrata* accounted for more than half of the cases of non-*albicans* VVC. Nevertheless, the recovery rate of *C. glabrata* (19.2%) in the present study was lower, compared to that reported in previous studies [ 19 , 21 , 29 , 30 ].

As already mentioned in most epidemiologic studies worldwide, among *Candida* species, *C. albicans* and *C. glabrata* were the most common causes of VVC [ 6 , 16 , 20 - 22 , 30 ]. However, some studies reported that *C. tropicalis*, *C. parapsilosis*, and *C. krusei* were the second most common cause of vaginal infection [ 3 , 4 , 18 , 35 ]. Notably, *K. marxianus*, *C. lusitaniae*, *C. inconspicua*, *C. guilliermondii*, and *C. dubliniensis* were previously reported as a rare and unusual cause of VVC by several investigators [ 4 , 16 , 19 , 27 , 35 - 37 ].

Along with these findings, we recovered emerging species of *Candida* from VVC patients, such as *C. fabianii* and *C. africana*. It seems that the widespread use of antifungal agents inappropriately has contributed to the emergence of such more azole-resistant non-albicans *Candida* species [ 38 , 39 ].

Similar to other studies, co-isolation with two species of *Candida* isolated from one sample was seen in 4.8% (n=6) of our VVC patients, all of which were *C. albicans* and *C. glabrata*. Mixed infection of *C. albicans* and *C. glabrata* with prevalence rates of 1.2% and 4.4% were reported by Maraki *et al*. and Gharaghani *et al*. respectively [ 6 , 30 ]. Despite our study, these investigators also reported co-isolation with two species of *C. albicans* and *P. kudriavzevii*, *C. glabrata*, and *C. tropicalis* and *C. albicans* and *K. marxianus* [ 6 , 30 ].

Since correct identification of different *Candida* species using rapid and reliable molecular tests in mixed infection can contribute to both successful treatment and better knowledge in species distribution, laboratories should be able to correctly identify these species in mixed infection [ 40 - 42 ]. MALDI-TOF MS is a strong technique to definitively identify emerging or cryptic *Candida* species that exhibit resistance patterns to the few available antifungal agents. Nevertheless, identification using MALDI-TOF MS is expensive, usually not available in routine clinical mycology laboratories, and requires highly expert and trained personnel.

Given that the development of antifungal drugs resistance in *Candida* isolates is increasing, the assessment of the antifungal resistance pattern against *Candida* species isolated from clinical samples is becoming increasingly important to choose the proper antifungal drug for the management of candidiasis [ 45 - 43 ].

In this study, the antifungal susceptibility profile of all Candida isolates was inquired against four azole drugs. As shown in Table 1, fluconazole (the recommended drug of choice for VVC) was susceptible against 94.2% (80/85) *C. albicans* isolates. Of note, 2.3% (2/85) of the *C. albicans* isolates were SDD and 3.5% (3/85) of them were resistant to fluconazole, which is in concordance with the findings of Maraki *et al*. [ 6 ].

The overall resistance rate of *Candida* species in our study against fluconazole was 3.2% (4/125). However, the higher overall resistance rate on fluconazole against *Candida* species isolated from VVC was reported by Bitew et al. [ 4 ] and Adjapong et al. [ 46 ] (17.2% and 26.6%, respectively). Regarding non-*albicans Candida* species, our study demonstrated that 32.5% (13/40) of non-*albicans Candida* species exhibited high MIC values (>4) to fluconazole except for *P. kudriavzevii*. Generally, we observed only 5% (2/40) fluconazole resistance among non-*albicans* Candida species in our study. 

In contrast to our study, results of earlier studies in Greece and Ghana indicated the high fluconazole resistance (15.6% and 31.9%, respectively) against non-*albicans Candida* species isolated from VVC [ 6 , 46 ]. Resistance to voriconazole was observed only among vaginal *C. albicans* isolates 0.8% (1/125), while all other vaginal *Candida* species were susceptible or SDD to voriconazole. Maraki *et al*. [ 6 ] presented similar rates of resistance to voriconazole (1.6%) against all *Candida* species causing VVC. However, this is much lower, compared to the findings of a previously reported study by Adjapong et al. [ 46 ] that reported rates of up to 10.8%. Nejat *et al*. and Hasanvand *et al*. reported that itraconazole (MIC50 / 90, 0.063/0.125 μg/mL) was active against all *Candida* species [ 21 , 36 ].

In our study, the resistance rate to itraconazole was 4.7% (n=4) among *C. albicans* isolates. The overall resistance rate of *Candida* species in our study against itraconazole was 7.2% (9/125). The study of Adjapong *et al*. showed that the resistance rate to itraconazole was 10% among *C. albicans* isolates [ 46 ]. While resistance rate to itraconazole in a study conducted by Khan et al. was higher at 40.7% against all vaginal *Candida* isolates [ 47 ].

## Conclusion

Results of this study suggested that clinical isolates of *Candida* species need to be properly identified and their antifungal susceptibility profiles were determined to help clinicians to choose an appropriate antifungal drug with the high outcome of successfully treating VVC.

## Acknowledgement

This research was financially supported by the Fasa University of Medical Sciences in Fasa, Iran (ethical code: IR.FUMS.REC.1395.85).

## Authors’ contribution

M.H A. conceived the study. M.H A., R. K., and F. M. prepared the strains. M.H. A., K. A., and F. M. performed experiments. N. A. and M.H A. prepared the manuscript. N. A., M.H A., and N. V. analyzed the data and edited the final article. All authors read and approved the final manuscript. 

## Conflict of Interest

The authors declare that they do not have anything to disclose regarding funding or conflict of interest concerning this manuscript.

## Financial disclosure

No financial interests related to the material of this manuscript have been declared.
